# Are non-attenders a concern for primary care practice?

**DOI:** 10.1186/2045-4015-2-13

**Published:** 2013-03-27

**Authors:** Vladimir Khanassov, Isabelle Vedel, Howard Bergman

**Affiliations:** 1 Department of Family Medicine, McGill University, 517 Pine Avenue West, Montreal, Quebec, H2W 1S4, Canada

## Abstract

The article by Eshel *et al.* describes major differences, in terms of demography and health status, between elderly patients who did and those who did not visit primary care physicians for general health check-ups. The authors conclude that non-attenders are not at risk for developing health conditions.

While this study by Eshel *et al.* provides a better understanding of the primary care population, the conclusion (no need for reaching out to the non-attenders) should be viewed with caution. In this study, non-attenders ‘have a higher probability of being women, older, not married and from a lower socio-economic’ segment of the population, a population that is known to be at higher risk for chronic disease. In addition, outreach programs in primary care would be key in providing essential preventive measures for this vulnerable population (e.g., osteoporosis prevention, vaccination, lifestyle, etc.).

This is a commentary on http://www.ijhpr.org/content/2/1/7.

## 

The study of Eshel *et al.* touches on the importance of the need to develop outreach programs for a population that is unable or reluctant to have regular annual check-ups with their primary care physician [1]. The authors explore the assumption that this group of patients (non-attenders) would not benefit from a general health check-up at least once a year. This study is the first since the late 1990s to characterize the demography and health status of elderly patients who had not seen their primary care physician for at least two years (2007 – 2009). This group of Israeli patients had fewer hospitalizations and visits to specialists, and less prescribed medications and laboratory procedures (e.g., blood tests and imaging).

The study findings confirm results of studies conducted in the late 1990s in that a decision not to see a primary care physician is related to better health and may be an expression of autonomy. For example, several studies examined this group of infrequently attending patients and concluded that in the majority of cases, they do not suffer from serious conditions requiring medical attention [2-5]. Based on primary care patient interviews, Thomas *et al.* showed that they have higher scores on perceived health dimensions such as physical, role and social functions, mental health, health perception, and pain (p<0.01 for each) [2]. In addition, study results showed that people over 85 years of age who do not consult their primary care physician, are in a better emotional state [6], and have a large support network of family and friends [7]. These results are thus in line with those by Eshel *et al.* Moreover, service use (e.g., emergency department) is lower in the group of non-attenders [2,3]. However, not seeing a primary care physician is also linked to a lower use of preventive services (e.g., lower rate of cervical smears, mammography, and visits to the dentist) [2,6] that can lead to adverse consequences requiring care and services in the long-run. The most recent study by Suominen-Taipale *et al.* is based on cross-sectional surveys conducted in two countries with public health care systems (Norway and Finland) [8]. The study demonstrated that poor self-rated health was strongly associated with the use of specialized care. In addition, their higher education predisposes elderly Finns to visit a specialist more often than a general practitioner.

While this very interesting study by Eshel *et al.* provides a better understanding of the primary care population, the conclusion (no need for reaching out to the non-attenders) should be viewed with caution. In this study, non-attenders ‘have a higher probability of being women, older, not married, and from a lower socio-economic’ segment of the population. This population is known to be at higher risk for chronic disease, and for its inappropriate underutilization of health care resources, in particular health promotion and preventive measures [9]. The authors recognize that the lower rate of chronic disease may be an artifact in this category of patients. As well, the researchers do not estimate functional status and mortality rate, making it difficult to evaluate and compare the severity of chronic diseases between the two groups. Thus, the characteristics of the non-attenders should be taken into account when policymakers develop the outreach programs.

Even if it is assumed that the elderly population who receive care from a public health care system are in better health, one of the objectives of primary care is to help patients maintain this status. Outreach programs would be key in providing essential preventive measures for this vulnerable population (e.g., osteoporosis prevention, vaccination, lifestyle, etc.) [10].

### Competing interests

The authors declare that they have no competing interests.

### Authors’ information

Vladimir Khanassov, MD, MSc Candidate, Department of Family Medicine, McGill University

Isabelle Vedel, MD, PhD, Assistant Professor, Department of Family Medicine, Division of Geriatrics, McGill University

Howard Bergman, MD, FCFP, FRCPC, Chair of the Department of Family Medicine, Professor of Family Medicine, Medicine and Oncology, The Dr. Joseph Kaufmann Professor of Geriatric Medicine, McGill University
